# A rare case of sphenoid sinus lymphoma

**DOI:** 10.1002/ccr3.6585

**Published:** 2022-11-13

**Authors:** Brian L. Risavi, Kevin Elwell, Courtney Whiteman

**Affiliations:** ^1^ Lake Erie College of Osteopathic Medicine Erie Pennsylvania USA; ^2^ UPMC Hamot Erie Pennsylvania USA

**Keywords:** headache, lymphoma, sphenoid sinus

## Abstract

Malignancy of the sphenoid sinus is rare. Tumors may extend to adjacent anatomic structures before detection and may be easily missed. Outcomes are typically poor and vary by tumor type. Clinicians should maintain vigilance for neoplastic disease in patients presenting with headache and ocular/neurological complaints of the face/sinuses.

## 1 INTRODUCTION

Malignancy of the sphenoid sinus is rare, representing <1% of all cancers.^1^ The most common types are squamous cell, adenocarcinoma, non‐Hodgkin's mature B‐cell lymphoma, and unspecified epithelial neoplasms.^1^ Tumors may extend to adjacent anatomic sites before detection.^2^ Each tumor may present with a broad range of symptoms, such as chronic sinusitis, and may be easily missed.^3^ Outcomes are typically poor.^1^ Survival rates, however, vary by type of tumor.^4^ The most common age group affected is 50–59, predominantly white males.^1^ Average tumor size is 3.7 cm.^1^

## 2 CASE

**FIGURE 1:**
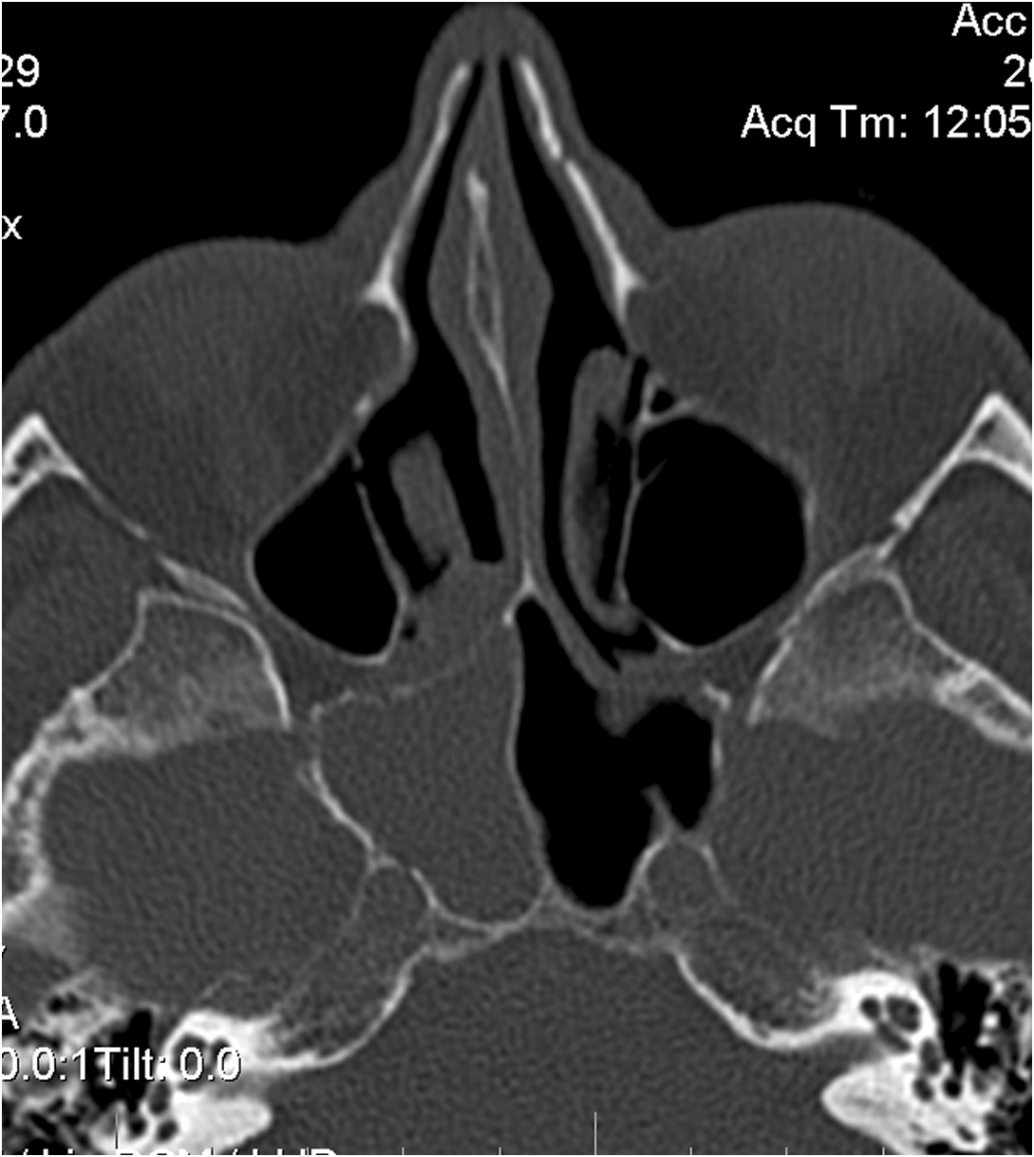
CT image of mass in sphenoid sinus transverse

**FIGURE 2:**
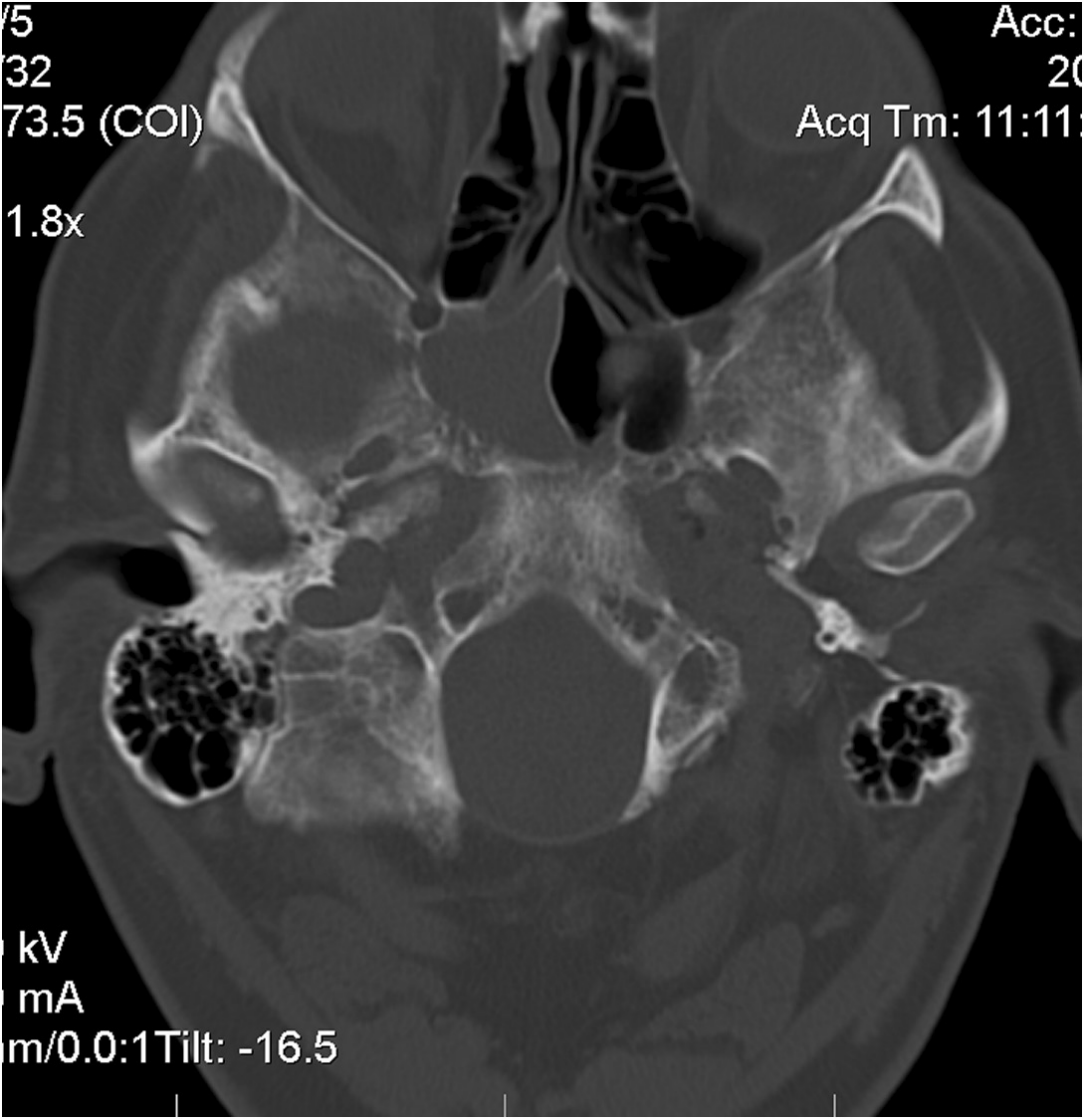
CT image of mass in sphenoid sinus transverse

**FIGURE 3:**
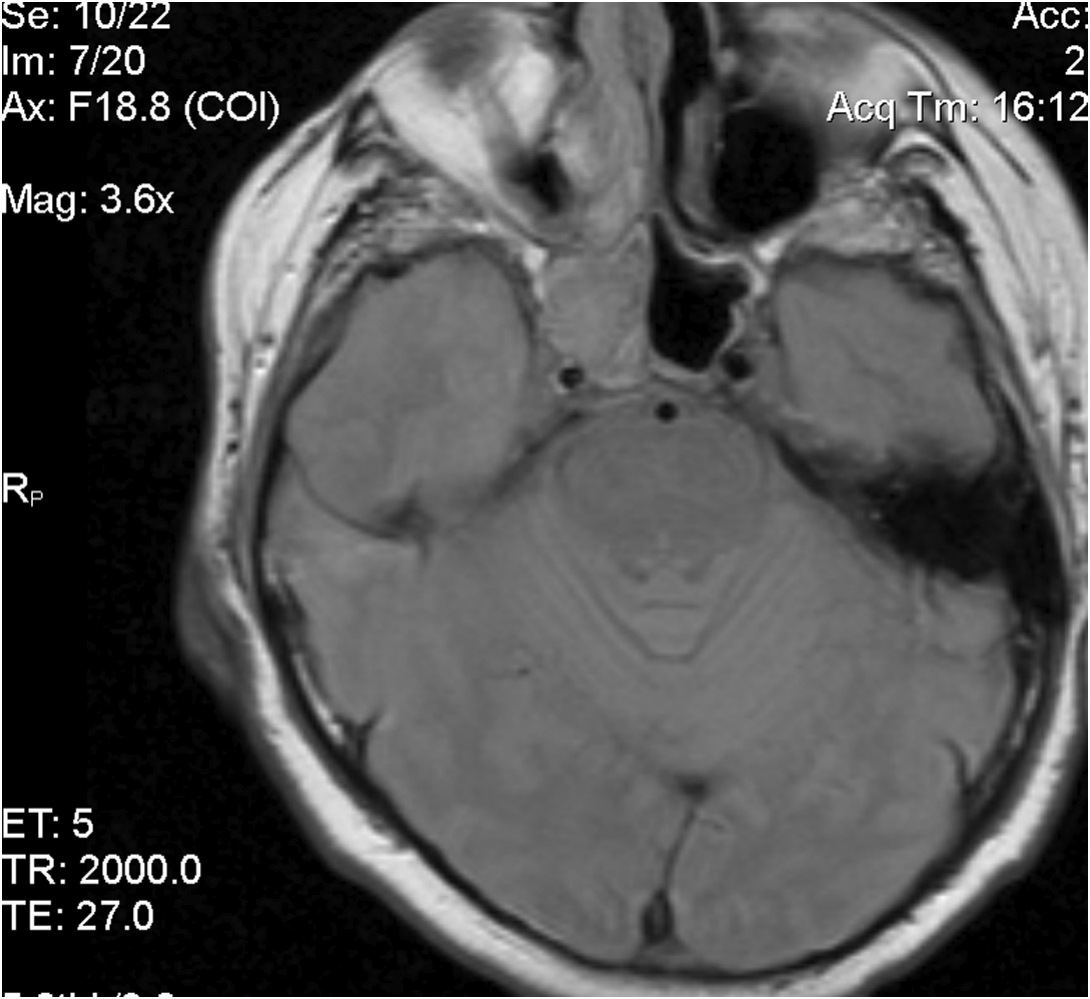
MRI image of mass in sphenoid sinus/optic nerve involvement transverse

**FIGURE 4:**
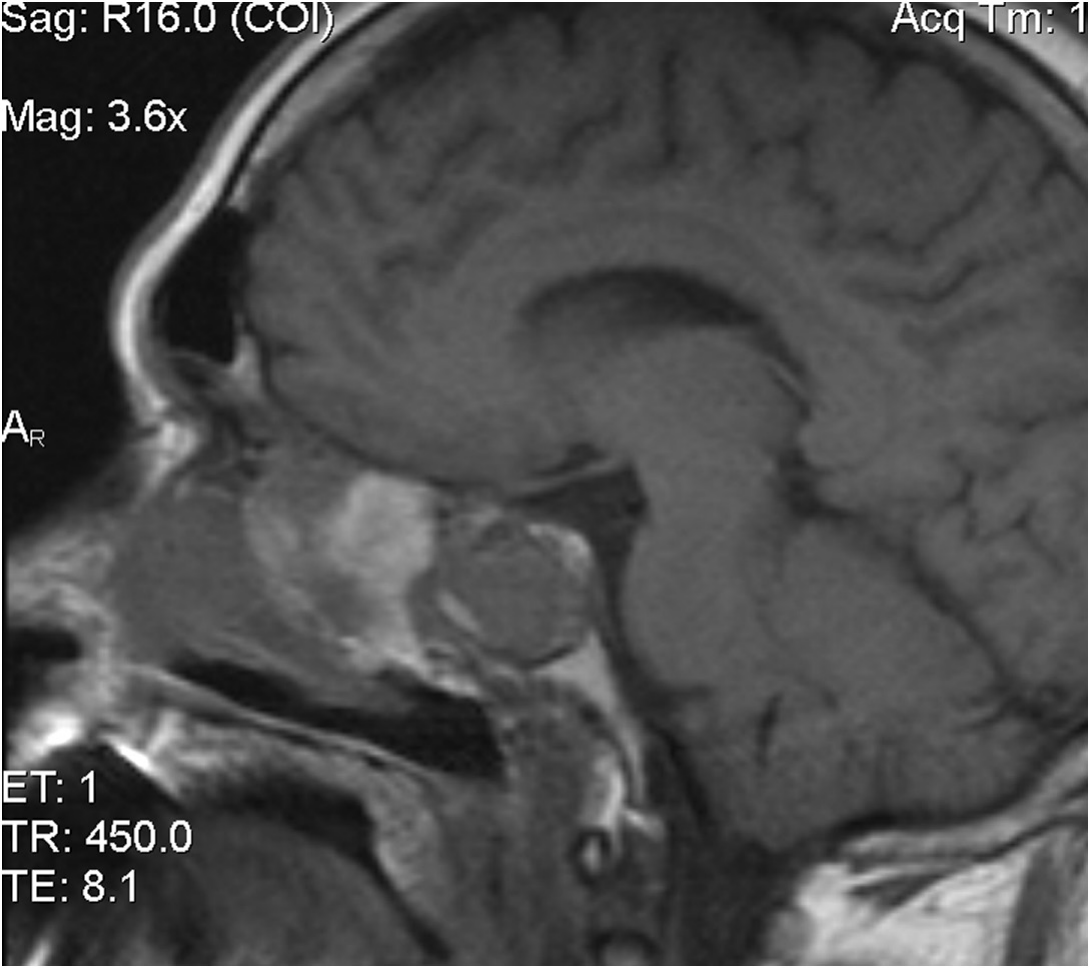
MRI image of mass in sphenoid sinus/optic nerve involvement sagittal

**FIGURE 5:**
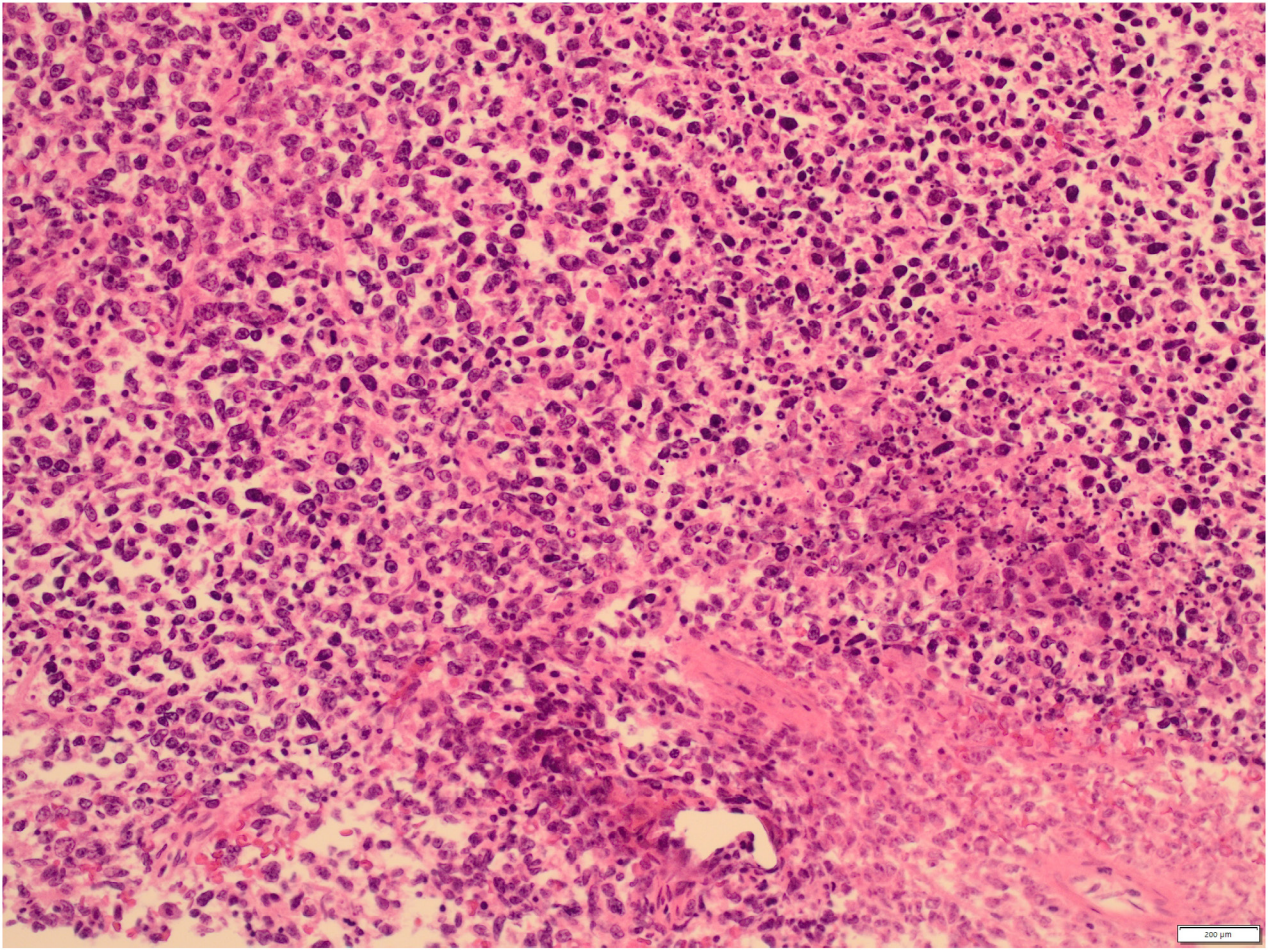
Hematoxylin and eosin stain of sphenoid sinus lymphoma 200×

**FIGURE 6:**
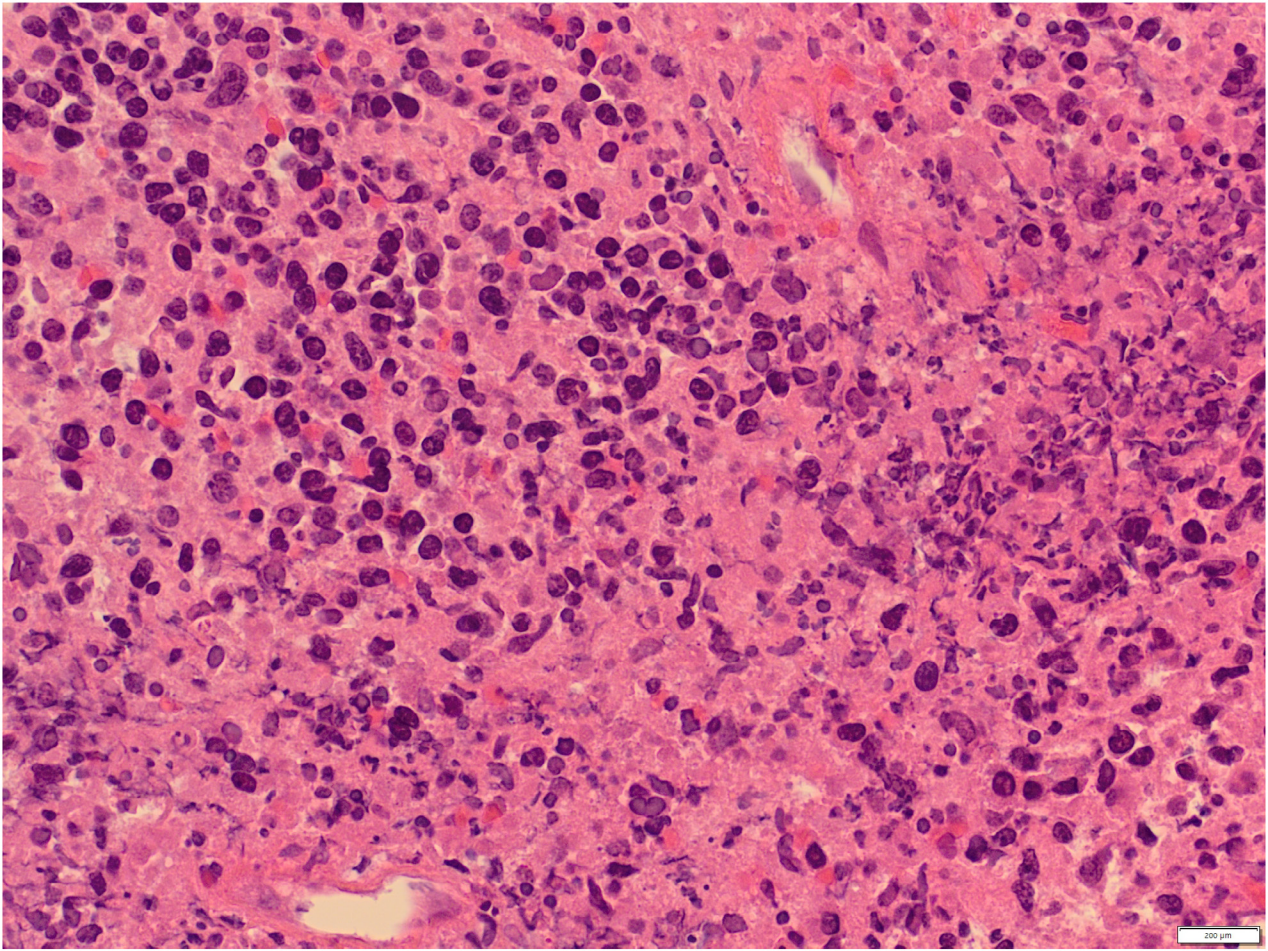
Hematoxylin and eosin stain of sphenoid sinus lymphoma 400×

**FIGURE 7:**
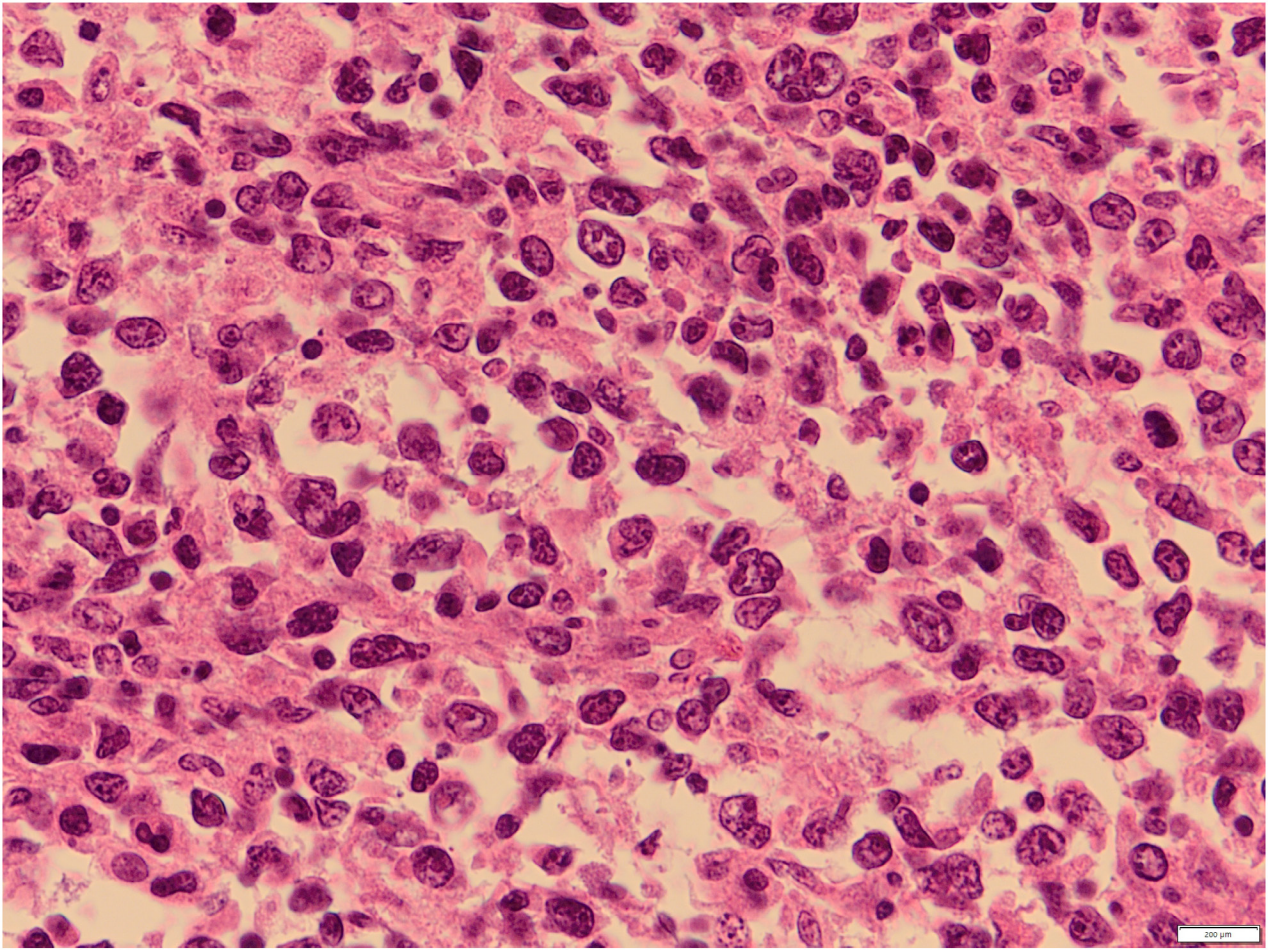
Hematoxylin and eosin stain of sphenoid sinus lymphoma 600×

A 54‐year‐old white male complained of an intermittent, increasingly severe, headache for 2 weeks. On the day of presentation to the emergency department, he awakened at 0600 with increasingly severe right periorbital pain, described as sharp, radiating down his right cheek and the right side of his neck. He had two episodes of nausea/vomiting. He denied any syncope, visual changes, vertigo, slurred speech, extremity weakness/paresthesias, fever/chills, or trauma. Review of systems was otherwise negative. Past medical history includes diabetes mellitus, migraine headaches, and Meniere's disease. He denied any tobacco, alcohol, or drug use. Family history includes migraines. On examination, the patient was awake/alert with stable vital signs. Patient was afebrile. HEENT examination revealed pupils to be equal/round/reactive to light. Photophobia was noted in the right eye. Extraocular muscles intact. No cranial nerve deficits. No nasal drainage. Tenderness over the right temporal artery noted. Uvula midline. No tongue deviation on protrusion. No evidence of carotid bruits noted. No cervical adenopathy or nuchal rigidity. The remainder of the examination was unremarkable. Diagnostic results revealed a white cell count of 4.0 and an ESR of 2. Lumbar puncture was negative. CT of the head revealed complete opacification of the sphenoid sinus (Figures 1 and 2). CT angiogram of the head revealed no abnormalities. Patient was admitted for further work‐up, antibiotics, and ENT consultation. Patient initially refused an MRI; however, he ultimately consented to an MRI of the brain which was negative. MRA demonstrated stenosis of the right anterior cerebral artery and right middle cerebral artery. Two days later, he developed a third nerve palsy and diplopia in the right eye secondary to optic nerve involvement. Biopsy of the sphenoid sinus revealed a large B‐cell lymphoma, which was wrapped around the optic nerve (Figures 3, 4, 5, 6, 7).

Patient treatment consisted of 6 months of chemotherapy with negative PET scans on follow‐up. Approximately 5 years thereafter, the patient was diagnosed with a GI bleed, fatty liver, and esophageal varices (for which banding was completed). Further evaluation revealed malignant hepatic lesions and metastasis to the spine causing back pain. Palliative radiotherapy was initiated but the patient passed shortly thereafter prior to further work‐up of the hepatic lesions.

## 3 DISCUSSION

Primary B‐cell lymphoma of the sphenoid sinus is very rare, having an incidence of 0.03 per 100,000 persons between 2000 and 2012.^1^, ^2^, ^5^, ^6^ Male predominance occurs approximately 2:1 with the majority occurring in patients >55 years of age, and more prominent in whites than blacks.^1^, ^7^ B‐cell lymphomas remain the most common non‐epithelial tumors of the paranasal sinuses.^2^, ^8^ The sphenoid sinus, contained within the confines of the sphenoid bone, is anatomically contiguous with the carotid arteries, optic nerves, the maxillary division of cranial nerve V, brain stem, and sella turcica.^3^ It does not arise as an invagination of the nasal cavity.^4^, ^7^ Consequently, clinical presentations vary widely, and occur in advanced stages due to local anatomy.^5^, ^7^, ^8^ B‐cell lymphomas frequently demonstrate soft tissue fossa destruction, especially in the orbit with associated proptosis.^9^ Complications of sphenoid sinus neoplasms include orbital apex syndrome (including visual impairment and extraocular muscle palsy), superior orbital fissure syndrome, optic neuritis, blindness, cavernous sinus thrombosis, meningitis, among others.^10^, ^11^ Headaches, often in the temporal, retroorbital, and retrobulbar regions, cranial neuropathies (cranial nerve VII most commonly and associated with worse prognosis), and facial pain/paresthesias may all occur.^3^, ^12^, ^13^, ^14^ Headaches are thought to be secondary to tumor effect on the sphenopalatine ganglion and remain the most common presenting symptom.^4^ Other common presenting symptoms include visual changes, and cranial nerve palsies.^12^, ^15^ The anatomic location makes resection challenging due to adjacent structures.^1^, ^4^ In our case, the tumor surrounded the optic nerve, but this is rare (Figures 3 and 4).^16^ Some cases may mimic Tolosa‐Hunt syndrome, a benign pathology consisting of unilateral orbital pain and nerve palsies of cranial nerves III, IV, and VI.^17^ Imaging often reveals opacification of the sphenoid sinus.^4^, ^5^ CT and MRI remain the imaging modalities of choice.^9^, ^14^ Biopsy is accomplished via endoscopy rather than resection for improved cosmetic outcome.^3^, ^7^, ^15^ Fortunately, B‐cell lymphomas are rapidly growing and thus very sensitive to chemotherapy and radiotherapy.^5^ Radiotherapy, however, is not without complications. Patients may experience panhypopituitarism as well as visual effects.^7^ Intensity‐modified radiation therapy may be utilized. In the eye, hyperfractionation is used rather than intensity‐modulated radiation resulting in a lower complication rate secondary to decreased radiation exposure to the eye.^7^ Combination chemoradiation continues to be the selected treatment of choice. The most common chemotherapy regimens include CHOP (cyclophosphamide, doxorubicin, vincristine, prednisolone) and occasionally BACOP (bleomycin, doxorubicin, cyclophosphamide, vincristine, prednisolone) or CEOP (cyclophosphamide, epidoxorubicin, vincristine, prednisolone).^2^ Rituximab is frequently supplemented to the CHOP regimen with significant improvement in survival especially among women.^2^, ^18^ Surgery may be possible in select cases such as a focal tumor with clear margins or if the patient is symptomatic secondary to tumor burden.^1^, ^2^, ^4^ The majority of cases are not as responsive to surgery; however, when compared to a combination of chemotherapy and radiation.^2^ Prophylactic intrathecal chemotherapy may be utilized as there is a potential for recurrence in the CNS.^6^ Biopsy of sphenoid sinus opacification must be considered in chronic sinusitis or when other previously mentioned signs/symptoms occur.^5^, ^14^, ^19^, ^20^ Importantly, opacification of the sphenoid sinus does not exclude lymphoma.^5^ Clinicians should maintain a high degree of vigilance for neoplastic disease in patients presenting with headache and ocular/neurological complaints of the face/sinuses.

## AUTHOR CONTRIBUTIONS

**Brian L. Risavi:** Conceptualization; data curation; formal analysis; writing – original draft; writing – review and editing. **Kevin Elwell:** Formal analysis; writing – original draft; writing – review and editing. **Courtney Whiteman:** Formal analysis; writing – original draft; writing – review and editing.

## FUNDING INFORMATION

No funding was received for this publication.

## CONFLICT OF INTEREST

The authors report no conflict of interest.

## ETHICAL APPROVAL

I certify that this material has not been published elsewhere, either in whole or part, and is not under consideration for publication in any other journal. I have personally and actively been involved in substantive work leading to the revised manuscript and the authors will hold themselves jointly and individually responsible for its content.

## CONSENT

Written informed consent was obtained from the patient's wife to publish this report in accordance with the journal's patient consent policy.

## Data Availability

Data sharing is not applicable to this article as no new data were created or analyzed in this study.
